# Assumptions and Properties of Two-Level Nonparametric Item Response Theory Models

**DOI:** 10.1017/psy.2024.9

**Published:** 2025-01-03

**Authors:** Letty Koopman, Bonne J. H. Zijlstra, L. Andries van der Ark

**Affiliations:** 1 Nieuwenhuis Institute for Educational Research, Faculty of Behavioural and Social Sciences, University of Groningen, Grote Kruisstraat 2/1, 9712 TS Groningen, The Netherlands; 2 Research Institute Child Development and Education, Faculty of Social and Behavioural Sciences, Nieuwe Achtergracht 127, 1018 WS Amsterdam, The Netherlands

**Keywords:** conditional association, latent variable models, manifest invariant item ordering, manifest monotonicity, nonparametric item response theory

## Abstract

Nonparametric item response theory (IRT) models consist of assumptions that restrict the joint item-score distribution. These assumptions imply stochastic ordering properties that allow ordering of respondents and items using the simple sum score and item mean score, respectively, and imply observable data properties that are useful for investigating model fit. In this paper, we investigate these properties for two-level nonparametric IRT. We introduce four two-level nonparametric IRT models. Two models pertain to respondents nested in groups: The MHM-1, useful for ordering respondents and groups, and the DMM-1, useful for ordering respondents, groups, and items. Two models pertain to groups rated by multiple respondents: The MHM-2, useful for ordering groups, and the DMM-2, useful for ordering groups and items. We define the model assumptions, derive implied stochastic ordering properties, and derive observable data properties that are useful for model fit investigation. Relations between models and properties are also presented.

## 1 Introduction

Most item response theory (IRT) models implicitly assume that the respondents are a random sample from the population envisaged. These IRT models assume one or possibly more latent variables only at the level of the respondent, and we refer to these IRT models as single-level IRT models. However, in many practical situations, the respondents are nested in groups. For example, students nested in school classes rating their teacher’s instructional quality (Scherer et al., 2016), employees of the same department assessing humor in the workplace climate (Cann et al., 2014), or nurses within the same intensive care unit evaluating collaboration (Dougherty & Larson, 2010). In such situations, it is inappropriate to assume that the respondents are a random sample due to the group effect. It is therefore reasonable to use IRT models with a latent variable both on the respondent level and the group level (e.g., De Jong & Steenkamp, 2010; Fox, 2007; Fox & Glas, 2001). We refer to these IRT models as two-level IRT models. This paper investigates the measurement properties of a general nonparametric two-level IRT model, which was proposed by Snijders (2001), and which can be considered a two-level generalization of the single-level nonparametric IRT models proposed by Mokken (1969) and Holland and Rosenbaum (1986).

Assume that a test consists of *I* items, indexed by *i* ( 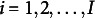 ), and each item has 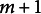 ordered item scores 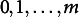 . Assume that this test is administered to *R* randomly selected non-nested respondents, indexed by *r* ( 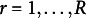 ). Note that index *r* refers to the *r*th respondent in the sample. Before sampling, it is not known which respondent from the population will be the *r*th respondent in the sample. Therefore, 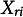 — defined as the score of the randomly selected *r*th respondent in the sample on item *i*—is a random variable. In this paper, variables will be denoted by uppercase letters, and their realizations by lower case letters. Hence, the realization of 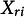 is denoted by 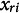 . For each respondent, the *I* item scores can be collected in a vector 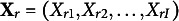 . Because the respondents are randomly and independently sampled, we consider the *R* vectors 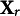 independent and identically distributed (i.i.d.) for all *r*. As the respondents are non-nested, a single-level IRT model may be appropriate as a measurement model. Let 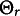 be a random latent variable of the *r*th randomly sampled respondent. Analogous to 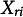 , 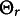 is a random variable, because before sampling it is not known which respondent from the population will be the *r*th respondent in the sample. Because the respondents are randomly and independently sampled, the *R* variables 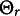 are i.i.d. for all *r*. Let 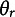 be a value of respondent *r* on the random latent variable 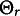 . For respondent *r*, the expected value on item *i* is 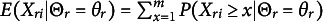 . The expectation of 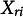 as a function of 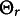 , 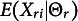 , is referred to as the item response function (IRF; Chang & Mazzeo, 1994). Most single-level IRT models are defined by at least these three assumptions:

1. Unidimensionality (UN): Latent variable 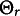 is unidimensional
2. Local independence (LI): Item scores 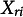 are independent given 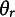

3. Monotonicity (MO): 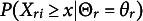 is nondecreasing in 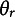 , for all *i* and for 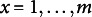

These assumptions are necessary to restrict the distribution of 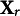 (Junker & Ellis, 1997). The combination of UN, LI, and MO is also referred to as the *monotone homogeneity model* (MHM, Mokken, 1971; Sijtsma & Molenaar, 2002; a.k.a. monotone unidimensional representation, Junker, 1993; Junker & Ellis, 1997; unidimensional monotone latent variable model, Holland & Rosenbaum, 1986; and nonparametric graded response model, Hemker et al., 1996, 1997). The MHM does not use parameters to model the distribution of 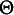 and the relation between the item scores and 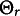 . The MHM is therefore called a nonparametric IRT model.

A fourth assumption in nonparametric IRT is invariant item ordering. Suppose that the *I* items are ordered by mean item score and numbered accordingly; that is, if 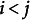 , then 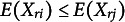 for all 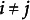 . Then,

1. Invariant item ordering (IIO): 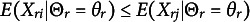 for all 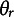

(Ligtvoet et al., 2011; Sijtsma & Hemker, 1998; Sijtsma & Junker, 1996). IIO means that the order in difficulty is identical across all values of the latent variable. IIO allows the stochastic ordering of the items using the mean item scores. For applications of IIO, we refer to Sijtsma et al. (2011). Following Sijtsma and Van der Ark (2017, 2020, pp. 156–158; also see the Discussion), we call the model that assumes UN, LI, MO, and IIO the double monotonicity model (DMM).

The MHM has several ordering properties. The MHM implies stochastic ordering of the manifest variable by the latent variable (Hemker et al., 1996, 1997), which implies that latent variable can be used stochastically to order the respondents on the unweighted sum score. More importantly, for dichotomous items, the MHM implies monotone likelihood ratio (MLR; Grayson, 1988; Huynh, 1994; Ünlü, 2008), which implies the property of stochastic ordering of the latent variable by the sum score across the items (SOL; Hemker et al., 1997). Measurement properties MLR and SOL imply that the sum score can be used to (stochastically) order respondents on the latent variable. For polytomous items, the MHM does not imply MLR and SOL (Hemker et al., 1996, 1997); however, the MHM implies the measurement property of weak SOL (Van der Ark & Bergsma, 2010), which can be used for pairwise ordering of respondents or groups on the latent variable.

These theoretical results justify ordinal person measurement by means of sum score 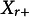 if the MHM holds. Suppose that two respondents have sum scores *a* and *b*, respectively ( 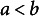 ), then for dichotomous items, due to the SOL property, the MHM implies 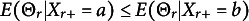 ; for polytomous items, due to the weak SOL property, the MHM implies 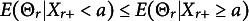 . Hence, the sum score stochastically orders the respondents on 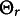 . Alternatively, suppose that two respondents have latent variable values *t* and *u*, respectively ( 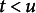 ), then due to the monotonicity assumption the MHM implies 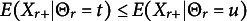 . Hence the latent variable values stochastically orders the respondents on the sum score, a property sometimes referred to a stochastic ordering of the manifest variable by 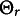 (SOM; Hemker et al., 1997). These mutual ordering properties of 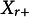 and 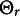 make 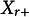 an attractive estimator of 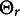 . Under the MHM, 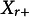 is a consistent asymptotic normal estimator of 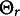 (Junker, 1991; Stout, 1990).

The simple sum score is more intuitive for non-psychometricians than, for example, an estimated latent variable because the sum score is defined on the scale of the test. Therefore, a higher sum score has a fairly straightforward interpretation, such as responded to more items correctly or responded more extreme to the items (Sijtsma & Hemker, 2000). In addition, using the sum score in scientific research avoids sample-specific transformations, which benefits comparability across studies and contributes to the replicability of results across studies (Edelsbrunner, 2022; Widaman & Revelle, 2022). Hence, providing justification for using the sum score is relevant for psychometric research and testing practice, even when the estimated latent variable is used for test construction and measurement evaluation (Hemker et al., 2001).

The DMM implies an ordinal scale for both person and item measurement. Hence, besides using the respondent sum score to order respondents on a latent variable, the mean item score can be used to order the items on a latent difficulty scale. Using the mean item score has similar advantages as the sum score for psychometric and testing practice: They have an intuitive interpretation, such as the proportion correct or average extremeness in the sample. In addition, estimating a latent difficulty is not straightforward and can have various interpretations that do not necessarily relate to the difficulty in practice (Sijtsma & Hemker, 2000; Sijtsma & Meijer, 2001).

All popular unidimensional IRT models, such as the Rasch Model (Rasch, 1960), the two- and three-parameter logistic models (Birnbaum, 1968), the graded response model (Samejima, 1969), the rating scale model (Andrich, 1978), the partial credit model (Masters, 1982), and the sequential model (Tutz, 1990), are special cases of the MHM (Van der Ark, 2001). Hence, if the goal of the test is to order respondents, the MHM is preferred over popular parametric IRT models because, by definition, the MHM fits better to the data than these parametric IRT models. If the goal of the test is estimating the respondents’ scores on 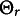 , alternative methods are required, such as a smoothing procedure or estimating a parametric IRT model (e.g., Ramsay, 1991; Sijtsma & Van der Ark, 2020, Chapter 4, respectively). However, as these parametric IRT models are a special case of the MHM, investigating the fit of the MHM is still useful because if the MHM does not fit, neither do the parametric IRT models.

The MHM poses testable restrictions on the data, referred to as *observable properties*. For example, the MHM implies nonnegative inter-item covariances (e.g., Sijtsma & Molenaar, 2002, pp. 155–156). Observable properties can be investigated in data to find evidence against the MHM assumptions. Holland and Rosenbaum (1986) showed that the MHM implies *conditional association* (CA). Let 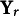 and 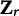 be two mutually exclusive and exhaustive subsets of 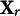 . CA holds if for every partitioning 
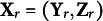
 and for all functions *h* and for all nondecreasing functions 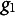 and 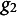


(1)

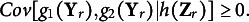



The observable property CA is too comprohensive for a single testing procedure (see Ellis & Sijtsma, 2023), but special cases of CA, including testing for nonnegative covariances, have been proposed to test the MHM. We focus on *manifest monotonicity* (MM; Sijtsma & Hemker, 2000) and a testing procedure to identify locally dependent item sets using three cases of CA (Straat et al., 2016). For dichotomous items, CA implies MM (Ligtvoet, 2022). Let 
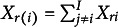
 be the rest score of item *i*, then MM means that (2)

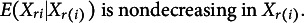

Hence, MM is the MO assumption with latent variable 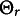 replaced by an observable proxy 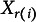 . Note that for polytomous items, the MHM does not imply MM. Straat et al. proposed testing 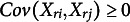 , 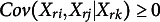 , and 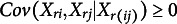 , where 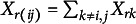 . We refer to these three inequalities as *nonnegative inter-item covariances* (NNIIC). As these three inequalities of NNIIC are special cases of CA with 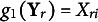 , with 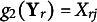 , and with 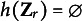 , 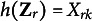 , and 
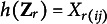
 , respectively, the MHM implies the three inequalities. Other CA-based observable properties have been proposed by, for example, Ellis (2014) and Ligtvoet (2022). Ellis and Sijtsma (2023) noted that these CA-based observable properties cannot distinguish between unidimensional and multidimensional models, and these authors suggested using (also CA-based) conditioning on added regression predictions (CARP) inequalities to investigate UN.

The DMM poses additional observable properties (see Ligtvoet et al., 2011). We focus on *manifest invariant item ordering* (MIIO), which holds if for 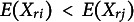 , (3)

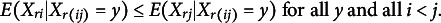

Note that MIIO is the IIO assumption with latent variable 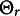 replaced by 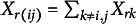 . Other observable properties of IIO have been proposed; for example, by Tijmstra et al. (2011).

The assumptions (UD, LI, MO, and IIO) discussed in this paragraph have not been formally defined for two-level IRT models, and as a result, it is also unknown how these assumptions should be investigated in test data. Also, the measurement properties MLR, SOL, and SOM nor the observable properties MM, CA, and MIIO have been defined for two-level IRT models, and as a result, it is unknown whether two-level IRT models imply these measurement properties in the same way as single-level IRT models do. In the remainder of this paper, we generalize the MHM and DMM to two-level data on both the respondent level and the group level. We build on the work of Snijders (2001), who proposed a two-level nonparametric IRT model for scaling subjects (e.g., persons or groups) scored by multiple respondents (i.e., multi-rater measurement) using dichotomous items. For the proposed models, we establish which stochastic ordering properties and observable data properties are implied, and how they are related. Note that the proofs have been diverted to the Appendix. Implications and recommendations for practice and further research are discussed.

## 2 Two-level nonparametric IRT

Suppose a measurement instrument consists of *I* items, indexed by *i* or *j* ( 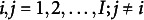 ). Suppose there are *S* groups, indexed by *s* ( 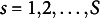 ), each consisting of 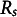 respondents, indexed by *r* 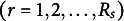 . Note that index *s* refers to the *s*th group, and index *r* refers to the *r*th respondent in group *s*. Before sampling, it is not known which group from the population of groups will be the *s*th group in the sample, nor which respondent from the population of respondents will be the *r*th respondent in group *s*. The groups are assumed to be a random sample from a population of groups, and the respondents within a group are assumed to be a random sample from a population of respondents. Without loss of generality, we assume the number of respondents per group is the same; that is, 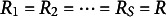 . Let 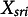 denote the score on item *i* of respondent *r* in group *s*, with realization 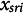 ( 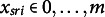 ). For dichotomous items, 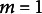 and 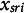 takes on value 1 if item *i* is endorsed or answered correctly by respondent *r* in group *s*, and 0 otherwise. Let 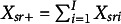 denote the respondent-level sum score. Let 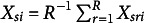 denote the group-level score on item *i* (i.e., the mean score over respondents’ scores on item *i* within group *s*), with realization 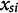 . 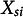 can take on 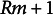 values with a minimum of 0 and a maximum of *m*. Let 
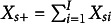
 denote the group-level sum score. The vector of item scores for respondent *r* in group *s* is denoted 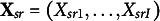 , with realization 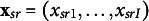 . Because the respondents within a group are randomly and independently sampled, the *R* vectors 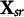 are considered i.i.d. within each *s* for all *r*. The vector of all item scores for group *s* is denoted 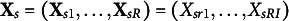 , with realization 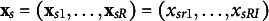 . Because the groups are randomly and independently sampled, the *S* vectors 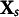 are considered i.i.d. for all *s*.

Let 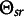 , 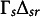 be random latent variables of the *r*th randomly sampled respondent in the *s*th randomly sampled group. Analogous to 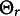 in the single-level situation, these are random variables because before the groups and respondents have been sampled, it is unknown which group from the population groups will be the *s*th group, and which respondent from the population of respondents belonging to the *s*th group will be the *r*th respondent. Variable 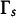 is considered a common group component, 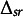 is a combination of an individual (random) respondent effect and a group by respondent interaction effect, and 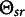 is the sum of these effects; that is, (4)

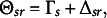

(Snijders, 2001). Let 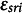 be a random latent variable that may be interpreted as an error term. Assumption 1 is a basic assumption(B) about the relation between the latent variables and the observed score 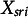 using function 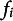 .

Assumption 1. Basic assumption of item scores and latent variables.

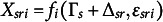
 . For all 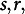 and *i*, 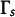 , 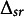 , and 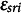 are independent. Furthermore, all 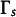 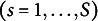 are identically distributed, and all 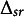 ( 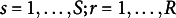 ) are identically distributed, with 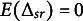 .

It follows from B that 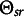 are identically distributed for all 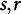 , and that for a fixed item *i*, all 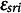 are identically distributed for all 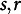 . B is assumed throughout this paper. The variances of 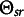 , 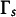 , and 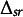 are denoted var( 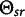 ), var( 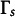 ), and var( 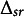 ), respectively. Because 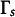 and 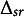 are assumed independent, 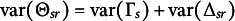 , for all *s* and all *r*. Let 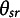 be a group-respondent combination value on 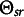 of respondent *r* in group *s*, 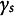 a value on 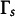 for group *s*, and 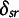 a value on 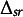 for respondent *r* in group *s*. Hence, for respondent *r* in group *s*, we assume there exist value 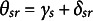 .

Let 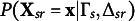 denote the probability of obtaining item-score pattern 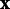 given 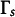 and 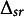 . Throughout the rest of the paper, we assume homogeneity of 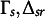 and 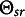 :

Assumption 2. Homogeneity assumption of 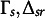 and 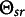

1. Homogeneity of the response probablities holds for 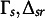 and 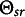 , hence, 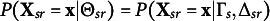

Let (5)

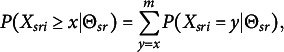

denote the probability of obtaining at least score *x* on item *i* given 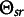 , which we refer to as the respondent-level item-step response function. For respondent *r* in group *s*, the expected item score is 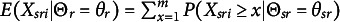 . In two-level test data, we distinguish between a respondent-level IRF (IRF-1, denoted 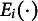 ) and a group-level IRF (IRF-2, denoted 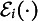 ). IRF-1 is defined as (6)

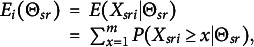

where 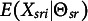 equals the expected item score 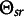 .

Let 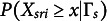 denote the probability of obtaining at least score *x* on item *i* given 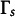 , which we refer to as the group-level item-step response function. By H and the law of total expectation (e.g., Rice, 2006, p 149), the item-step response function can be formulated as (7)

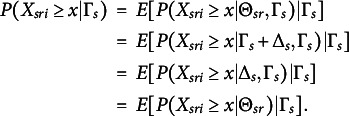

**Figure 1:**
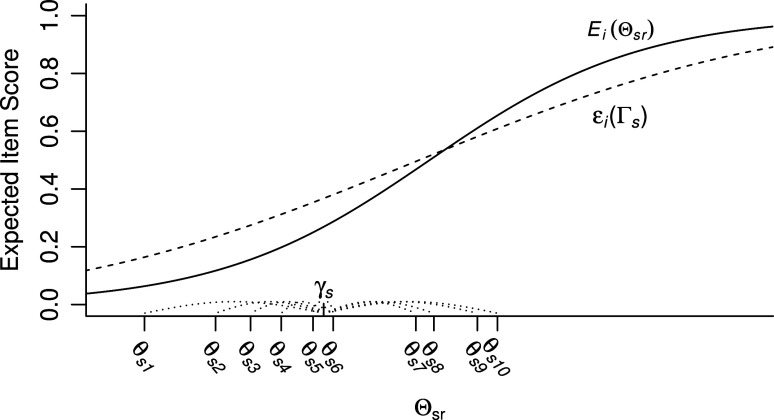
An IRF-1 ( 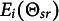 ; solid curve) and an IRF-2 ( 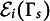 ; dashed curve) depicted on the same 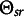 scale. The horizontal axis shows one hypothetical group value 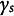 , plus the 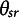 values of 10 randomly drawn respondents ( 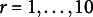 ) from group *s*. Note that 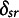 is represented by the length of the line segment between 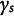 and the 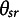 values on the horizontal axis.

For a randomly selected respondent in group *s*, the expected item score is 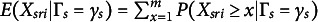 . IRF-2 is defined as (8)

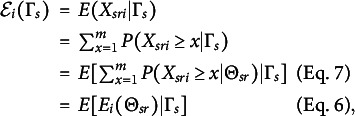

where 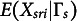 equals the expected item score as a function of 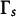 . Note that because 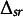 variables are assumed i.i.d., 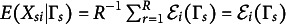 . Hence, the expected group-level item score for group *s* is the value of the IRF-2 for 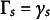 . Figure 1 shows an hypothetical IRF-1 and IRF-2. Because IRF-2 is the expectation of IRF-1 with respect to 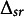 (Equation 8), IRF-2 is flatter than function IRF-1.

### 2.1 Definitions of possible model assumptions

Besides the basic and homogeneity assumption (B and H, respectively), multiple assumptions of nonparametric IRT for two-level data can be defined at level 1 (the respondent level) and at level 2 (the group level).

Definition 1. Unidimensionality (UN).

1. Unidimensionality at level 1 holds if 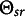 is a unidimensional variable.
2. Unidimensionality at level 2 holds if 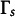 is a unidimensional variable.

UN-1 and UN-2 mean that the item scores on the test or questionnaire are modeled using one latent variable.

Definition 2. Local independence (LI).

- Local independence at level 1 holds if (9)

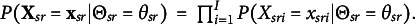

- Local independence at level 2 holds if (10)

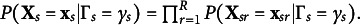

LI-1 means that respondent-level item scores ( 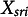 ) are independent given 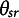 . LI-2 means that the response vectors of respondents are independent given 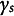 . LI-2 implies that between respondents, the respondent-level item scores 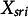 and 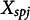 ( 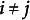 ; 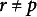 ) are independent given 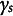 . However, within respondents, respondent-level item scores 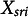 and 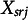 ( 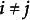 ) are not independent given 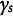 .

Definition 3. Monotonicity (MO).

1. Monotonicity at level 1 holds if 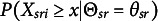 is nondecreasing in 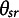 , for all *i* and 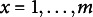 .
2. Monotonicity at level 2 holds if 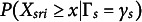 is nondecreasing in 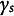 , for all *i* and 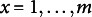 .

MO-1 implies that, for each item, IRF-1 (Equation 6) is nondecreasing in 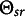 , and MO-2 implies that, for each item, IRF-2 (Equation 8) is nondecreasing in 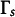 . Note that in Figure 1, IRF-1 satisfies MO-1 and IRF-2 satisfies MO-2.

Definition 4. Invariant item ordering (IIO). For a set of *I* items with 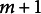 ordered item-score categories, for which the items are ordered and numbered such that 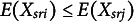 for all 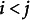 , then

- Invariant item ordering at level 1 holds if 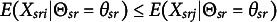 for all 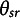 .
- Invariant item ordering at level 2 holds if 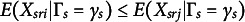 for all 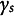 .

IIO-1 means that the IRF-1s of different items do not intersect. IIO-2 means that the IRF-2s of different items do not intersect. Note that the definition of IIO-1 and IIO-2 allows for ties, such that for some values of the latent variable items may be equally difficult.

### 2.2 Relation between level 1 and level 2 assumptions

Theorem 1 gives the relations between the basic assumption and local independence at both levels.

Theorem 1. B implies LI-1 and LI-2.

The assumptions UN, LI, MO, and IIO were defined at both Level 1 and Level 2 (Definitions 1 to 4). However, Theorem 1 shows that B implies both LI-1 and LI-2, and as a result, LI is no longer a necessary assumption, as in all remaining proofs LI-1 and LI-2 may be replaced by B and H.

Theorem 2 gives the relations between the assumptions at level 1 and the assumptions at level 2.

Theorem 2. Under B and H, UN-1, MO-1, and IIO-1 imply UN-2, MO-2, and IIO-2, respectively.

Theorem 2 shows that the level-1 assumptions imply their level-2 assumptions, but not the other way around. Hence, the level-2 assumptions do not imply the level-1 assumptions. For example, if respondent-level item scores depend both on 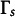 and on 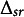 and var( 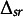 ) 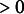 , in general 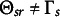 , 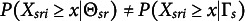 , and 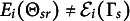 . As a result, UN-1, MO-1, and IIO-1 are not equal to UN-2, MO-2, and IIO-2, respectively. It may be noted that because of the homogeneity assumption H, the level-1 assumptions (UN-1, LI-1, MO-1, and IIO-1) are equivalent to the single-level nonparametric-IRT assumptions (UN, LI, MO, and IIO), when 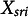 is replaced by 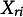 and 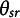 by 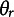 .

### 2.3 Models

Two-level nonparametric IRT assumptions can be used to define several nonparametric IRT models. Analogous to the single-level nonparametric IRT models, we distinguish between the MHM and the DMM, but in addition we also distinguish between the level on which they can be defined. Snijders (2001) defined a two-level nonparametric IRT model for scaling groups with dichotomous item scores using assumptions UN-1, LI-1, MO-1, and IIO-1. We present four models that allow for both dichotomous and polytomous items. As mentioned before, for all models B and H are assumed.

The first respondent-level model is the MHM-1, defined by assuming UN-1, LI-1, and MO-1 (Table 1, first row). The MHM-1 consists of level-1 assumptions, which imply UN-2, LI-2, and MO-2 (Theorem 2). The second respondent-level model is the DMM-1, defined by assuming UN-1, LI-1, MO-1, and IIO-1, implying UN-2, LI-2, MO-2, and IIO-2 (Table 1, second row). The first group-level model is the MHM-2, defined by assuming UN-2, LI-2, and MO-2 (Table 1, third row). The second group-level model is the DMM-2, defined by assuming UN-2, LI-2, MO-2, and IIO-2 (Table 1, fourth row). Note that, for all models, LI-1 and LI-2 are implied by B, but we explicitly incorporate them into the models, such that the models align more obviously to the single-level models.

**Table 1:** Assumptions of the two-level nonparametric IRT models

Model	Respondent-level assumptions				Group-level assumptions			
	UN-1	LI-1	MO-1	IIO-1	UN-2	LI-2	MO-2	IIO-2
MHM-1	A	A	A		I	I	I	
DMM-1	A	A	A	A	I	I	I	I
MHM-2					A	A	A	
DMM-2					A	A	A	A

*Note*: A = assumed, I = implied.

**Figure 2:**
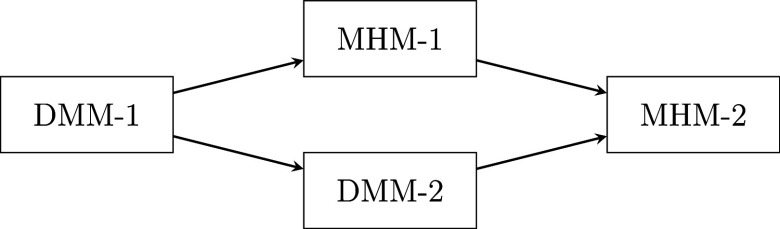
Hierarchical structure of the two-level nonparametric IRT models.

Figure 2 shows the hierarchical structure of the four models, where an arrow indicates an implication. The MHM-2 is the most general model, of which the other three are special cases. The DMM-1 is the most restrictive model, implying the other three models. In the next sections, we derive some ordering and observable properties implied by these models.

## 3 Ordering properties of two-level nonparametric IRT models

We investigated four possible ordering properties for sum score 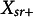 at level 1 and sum score 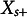 at level 2: MLR, SOM, SOL, and weak SOL.

Definition 5. Monotone likelihood ratio (MLR; Ferguson, 1967, p. 208).

1. Monotone likelihood ratio at level 1 holds if, for 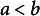 , the probability ratio (11)

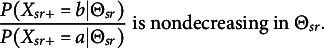

2. Monotone likelihood ratio at level 2 holds if, for 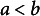 , the probability ratio (12)

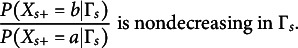

Definition 6. Stochastic ordering of the manifest score by the latent variable (SOM; Hemker et al., 1997).

1. Stochastic ordering of the manifest score by the latent variable at level 1 holds if, for any value *x* and 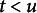


(13)

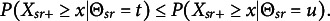

2. Stochastic ordering of the manifest score by the latent variable at level 2 holds if, for any value *x* and 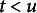


(14)

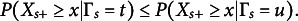

Definition 7. Stochastic ordering of the latent variable by the manifest score (SOL; Hemker et al., 1997).

1. Stochastic ordering of the latent variable by the manifest score at level 1 holds if, for any value *t* and 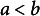 , (15)

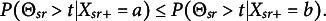

2. Stochastic ordering of the latent variable by the manifest score at level 2 holds if, for any value *t* and 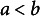 , (16)

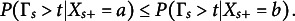

Definition 8. Weak SOL (WSOL; Van der Ark & Bergsma, 2010).

1. Weak SOL at level 1 holds if, for any *t* and *a*
(17)

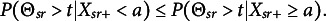

2. Weak SOL at level 2 holds if, for any *t* and *a*
(18)

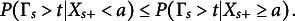

In general, ordering property MLR implies SOM, SOL, and WSOL, and SOL implies WSOL (Hemker et al., 1997; Lehmann, 1986, p. 85; Van der Ark & Bergsma, 2010). Hence, ordering property MLR-1 implies SOM-1, SOL-1, and WSOL-1, whereas ordering property MLR-2 implies SOM-2, SOL-2, and WSOL-2. The MLR, SOM, SOL, and WSOL results are valid for any monotone nondecreasing item summary within respondents (e.g., all-correct score, rest-scores, subscores; Rosenbaum, 1984).

For two-level test data, it is unknown whether MLR, SOM, SOL, or WSOL are implied by the two-level nonparametric IRT models. Theorem 3 gives the result for the strongest ordering property (MLR) for the least restrictive models (MHM-1 and MHM-2) and generalizes to weaker ordering properties and more restrictive models.

Theorem 3.

- For dichotomous item scores, the MHM-1 implies MLR-1.
- For dichotomous item scores, for 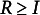 , the MHM-2 implies MLR-2.

MLR is symmetric in its argument, so the statement 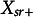 has MLR in 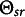 means that 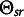 also has MLR in 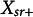 . Theorem 3 implies that for dichotomous items, under the MHM-1 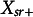 is stochastically ordered by 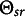 (SOM-1) and 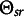 is stochastically ordered by 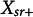 (SOL-1). It may be noted that Theorem 3(a) is very similar to the result obtained by Grayson (1988) who proved for single-level dichotomous item scores that the MHM implies MLR. Under the MHM-2, for 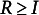 , group-level item score 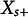 is stochastically ordered by 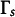 (SOM-2) and 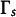 is stochastically ordered by 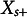 (SOL-2). Note that for 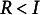 , MLR-2 is implied for the sum score of any random subset of items of size 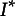 , for which 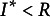 . Because the DMM-1 is a special case of the MHM-1 (see Figure 2), Theorem 3(a) also applies to the DMM-1. Similarly, the MHM-1, the DMM-1, and the DMM-2 are special cases of the MHM-2 (see Figure 2), Theorem 3(b) applies to these models as well.

For polytomous items, the single-level MHM and DMM generally do not imply MLR and SOL (see Hemker et al., 2001, for counter examples), but these models do imply SOM (Hemker et al., 1996, 1997) and weak SOL (Van der Ark & Bergsma, 2010). Theorem 4 and 5 show that these results generalize to two-level models.

Theorem 4.

- The MHM-1 implies SOM-1.
- The MHM-2 implies SOM-2.

Theorem 4 implies that under the MHM-1 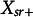 is stochastically ordered by 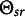 (SOM-1) and under the MHM-2, 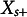 is stochastically ordered by 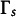 (SOM-2). Because the DMM-1 is a special case of the MHM-1, it also implies SOM-1. Also, because the MHM-1, the DMM-1, and the DMM-2 are special cases of MHM-2, these models imply SOM-2.

Theorem 5.

- The MHM-1 implies WSOL-1.
- The MHM-2 implies WSOL-2.

Let 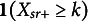 denote the dichotomized respondent-level sum score that takes on value 1 if 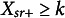 , and 0 otherwise, and let 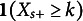 denote the dichotomized group-level sum score that takes on value 1 if 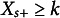 , and 0 otherwise. Then, Theorem 5 implies that under the MHM-1, 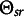 is stochastically ordered by 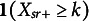 (WSOL-1), and that under the MHM-2, 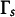 is stochastically ordered by 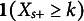 (WSOL-2). Because DMM-1 is a special case of MHM-1, this model also implies WSOL-1. Also, because the MHM-1, the DMM-1, and the DMM-2 are special cases of the MHM-2, these models imply WSOL-2.

## 4 Observable properties of two-level nonparametric IRT models

We define observable properties CA, MM, and MIIO for two-level IRT models. For single-level IRT models, rest score 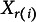 was used in MM, and rest score 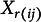 was used in NNIIC and in MIIO. These rest scores are proxies for the latent variable that must be independent of the variables under investigation. Because of the independence requirement, for two-level IRT models, these rest scores become more involved. Table 2 provides an overview of these rest scores for classified by observable property and level. The rest scores at Level 1 can be considered within-respondent rest scores, and the rest scores at Level 2 can be considered between-level rest scores.

**Table 2:** Overview of rest scores used in observable properties in single-level and two-level IRT models

Observable	Single-level IRT	Two-level IRT	
property		Level 1	Level 2
MM	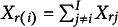	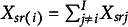	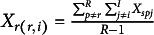
NNIIC 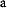	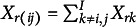	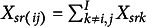	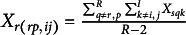
MIIO	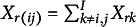	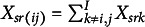	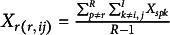

*Note*: ^a^ Pertains to the NNIIC given the rest score. The other two NNIIC inequalities do not use a rest score.

Definition 9 defines CA for two-level IRT models. First, partition 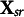 into two mutually exclusive and exhaustive sets 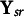 and 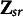 . For example, 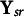 may contain 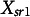 and 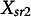 and 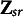 the remaining item scores. Second, partition the response vectors of the *R* respondents in group *s* — 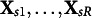 — which are collected in 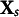 , into three mutually exclusive and exhaustive sets: 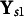 , 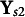 , and 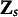 . For example, 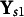 could contain just 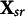 , 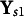 could contain just 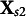 , and 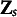 could contain the remaining response vectors from 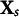 . Note that all scores of the same respondent are in the same set.

Definition 9. Conditional association (CA; Holland & Rosenbaum, 1986; Rosenbaum, 1988).

- Conditional association at level 1 holds if (19)

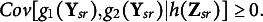

- Conditional association at level 2 holds if, for 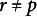 , (20)

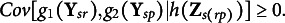

CA-1 is conditional association of the scores within respondents, whereas CA-2 is conditional association of the scores between respondents in the same group (see, also, Rosenbaum, 1988). As for CA, CA-1, and CA-2 are too comprehensive for a single test procedure. The testing procedure to identify locally dependent item sets using NNIIC (Straat et al., 2016) can be readily generalized to two-level models: For Level 1, the three inequalities in NNIIC generalize to 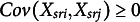 , 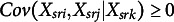 , and 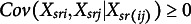 . For Level 2, let *p*, *q*, and *r* index three different respondents. The three inequalities generalize to 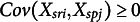 , 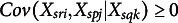 , and 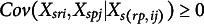 . Rest scores 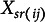 and 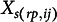 have been defined in Table 2.

Definition 10 defines MM for two-level IRT models. The rest scores used in Definition 10 have been defined in Table 2

Definition 10. Manifest monotonicity (MM; Junker, 1993; Sijtsma & Hemker, 2000).

1. Manifest monotonicity at level 1 holds if the within-respondent item-rest regression 
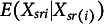
 is nondecreasing in 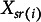 .
2. Manifest monotonicity at level 2 holds if the between-respondent item-rest regression 
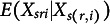
 is nondecreasing in 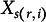 .

(first row)

Definition 11 defines MM for two-level IRT models. The rest scores used in Definition 11 have been defined in Table 2

Definition 11. Manifest invariant item ordering (MIIO; Ligtvoet et al., 2010).

- Manifest invariant item ordering at level 1 holds if, for 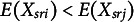 , 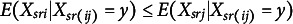 for all *y* and all 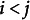 .
- Manifest invariant item ordering at level 2 holds if, for 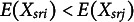 , 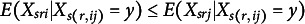 for all *y* and all 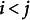 .

(second row).

In Theorem 7, 6, and 8, we state which two-level models imply the observable properties CA, MM, and MIIO, respectively.

Theorem 6.

- The MHM-1 implies CA-1.
- The MHM-2 implies CA-2.

Because the DMM-1 is a special case of the MHM-1, it also implies CA-1. Also, because the MHM-1, the DMM-1, and the DMM-2 are special cases of MHM-2, these models imply CA-2.

Theorem 7.

- For dichotomous items, the MHM-1 implies MM-1.
- For dichotomous items, the MHM-2 implies MM-2.

Because the DMM-1 is a special case of the MHM-1, it also implies MM-1. Also, because the MHM-1, the DMM-1, and the DMM-2 are special cases of MHM-2, these models imply MM-2. As for single-level IRT models, MM does not necessarily hold for polytomous items. However, MM-1 and MM-2 may still provide heuristic evidence for or against the MHM-1 and/or the MHM-2 (cf., Sijtsma & Van der Ark, 2020, p. 151). Alternatively, if polytomous items are dichotomized, Theorem 7 holds (Junker & Sijtsma, 2000).

Theorem 8.

- The DMM-1 implies MIIO-1.
- The DMM-2 implies MIIO-2.

Because the DMM-1 is a special case of the DMM-2, it also implies CA-2.

## 5 Relations between models and properties

In the previous sections, we defined four models, eight ordering properties, and six observable properties. In addition, we provided proofs for which model implied which property, for the least restrictive model and strongest property possible. Because more restrictive models are special cases of models with fewer restrictions, they are defined with at least the same assumptions that imply the property (see Figure 2). Hence, more restrictive models imply the same properties as the more general models.

Table 3 provides an overview of the most important implications for each model. The MHM-1 (Table 3, first column) implies (W)SOL-1 and (W)SOL-2. Hence, the MHM-1 implies an ordinal respondent-level scale, on which respondents may be stochastically ordered on 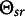 using 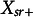 , and an ordinal group-level scale, on which groups may be stochastically ordered on 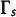 using 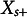 . Methods for investigating the model fit of the MHM-1 are MM-1, CA-1, MM-2, and CA-2. In addition to the implications by the MHM-1, the DMM-1 (Table 3, second column) also implies an ordinal item scale on which items may be stochastically ordered on their latent difficulty using the mean scores on the items. Methods MIIO-1 and MIIO-2 can be used for investigating model fit of the DMM-1 in addition to the methods of the MHM-1.

**Table 3:** Implied properties of the two-level nonparametric IRT models

Ordering property		Model		
	MHM-1	DMM-1	MHM-2	DMM-2
MLR-1	D	D		
SOL-1	D	D		
SOM-1	A	A		
WSOL-1	A	A		
MLR-2	D	D	D	D
SOL-2	D	D	D	D
SOM-2	A	A	A	A
WSOL-2	A	A	A	A
Observable property		Model		
	MHM-1	DMM-1	MHM-2	DMM-2
MM-1	D	D		
CA-1	A	A		
MIIO-1		A		
MM-2	D	D	D	D
CA-2	A	A	A	A
MIIO-2		A		A

*Note*: A = property is implied for dichotomous and polytomous items, D = property is implied for dichotomous or dichotomized items only.

The MHM-2 (Table 3, third column) implies (W)SOL-2. Hence, the MHM-2 implies an ordinal group-level scale on which groups may be stochastically ordered on 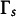 using 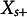 . Methods for investigating the model fit of the MHM-2 are MM-2 and CA-2. In addition to the implications by the MHM-2, the DMM-2 (Table 3, fourth column) also implies an ordinal item scale on which items may be stochastically ordered on their latent difficulty using the mean scores on the items. Methods MIIO-1 and MIIO-2 can be used for investigating model fit of the DMM-2 in addition to the methods of the MHM-2.

The two-level nonparametric IRT models are defined on either or both the respondent level and the group level. Depending on the interest of the researcher, one or both levels are relevant for scaling. If the goal is to scale the respondents, it is sufficient to mainly focus on checking the respondent-level assumptions of the MHM-1 or DMM-1. If the goal is to only scale the groups, as is the case in multi-rater data, the group-level assumptions are of key interest. For example, if a group-level IRF is flat, an item does not discriminate between low and high values of 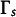 . Such an item does not contribute to accurate measurement on the group level. In addition, the respondent-level assumptions are informative for investigating, for example, whether the respondents may also be ordered using their sum score, or how the results relate to each other across levels. Therefore, even though investigating the MHM-2 or DMM-2 is sufficient to determine model fit at the group level, investigating the MHM-1 or the DMM-1 by checking assumptions on both level 1 and level 2 is suggested. If model violations occur at level 1, it is still possible that there are no violations at level 2, and the MHM-2 or the DMM-2 fits the data.

## 6 Discussion

The main contribution of this paper is the establishment of ordering properties and observable properties for two-level nonparametric IRT models. Ordering properties MLR-1, MLR-2, SOL-1.SOL-2, weak SOL-1, weak-SOL-2 SOM-1, and SOM-2 justify ordinal measurement using two-level nonparametric IRT models, in a way that is similar to ordinal measurement in the more popular single-level nonparametric IRT models. In addition, the observable properties MM-1, MM-2, CA-1, CA-2, MIIO-1, and MIIO-2 allow researchers to investigate the fit of the two-level nonparametric IRT models. Combined, these newly established ordering properties and observable properties enable the practical use of the two-level measurement models.

Building on previous work by Snijders (2001), we introduced four models for two-level test data. For level 1, we introduced the MHM-1, which allows ordering nested respondents on latent variable 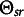 using manifest variable 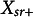 , and the DMM-1, which allows ordering nested respondents and items on 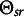 using 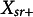 and 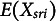 , respectively. For level 2, we introduced the MHM-2, which allows ordering groups on latent variable 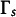 using manifest variable 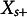 , and the DMM-2, which allows ordering groups and items on 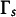 using 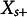 and 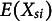 , respectively. The hierarchical relations among the four models show that the DMM-1 implies all other models and that the MHM-2 is the most general model (see Figure 2).

In addition, we derived observable data properties implied by the models, which can be used to investigate the model fit for a given data set. Specifically, we generalized the properties manifest monotonicity, conditional association, and manifest invariant item ordering for the respondent level and the group level. Theorem 7(b) showed the perhaps surprising result that, for a test consisting of dichotomous items, even though group-level item scores are not dichotomous (because they combine the item scores across respondents), still the strong results for dichotomous nonparametric IRT models hold. In deriving level-2 properties from level-1 properties, assuming the individual respondent-variables 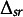 are i.i.d. proved to be a key ingredient. Assuming i.i.d. in test data is usually based on the sampling design or data collection conditions in relation to the latent variable. However, finding support for the i.i.d. assumption based on observable properties on the group level may be a valuable topic for future research.

The properties derived in this paper apply at the population level. Koopman et al. (2023) suggested statistical tests for MO-1, MO-2, IIO-1, and IIO-2 using observable properties MM-1, MM-2, MIIO-1, and MIIO-2, respectively. Using simulated data, these authors found that the tests for MO-1, IIO-1 and IIO-2 had satisfactory Type-1 error rates and power, whereas the tests for MO-2 had satisfactory Type-1 error rates but insufficient power (see also, Koopman, 2023). Note that both procedures deviated slightly from the results in this paper, because they used level-2 item scores rather than the between-respondent item scores that were used in the MM-2 and MIIO-2 definitions in this paper. Perhaps these latter item scores increases the power of the significance test of MO-2.

Note that Molenaar (1997) originally defined the DMM nonintersecting item-step response functions 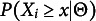 rather than an IRT model having nonintersecting item-response functions. As investigating properties of items can be considered more relevant than investigating properties of item-steps, the new definition of the DMM in terms of nonintersecting IRFs can be considered more useful. In addition, the property of IIO is defined in terms of conditional expected item scores and fits better to the new definition of the DMM than to the original definition. If there is reason to require an invariant item-step order, an alternative DMM-like model may be proposed including this assumption. However, one should realize that an invariant item-step order not necessarily implies an invariant item order (Sijtsma & Hemker, 1998).

In this paper, we chose to expand on work by Snijders (2001) because of its strong link to the one-level MHM and DMM. However, other generalizations of the MHM and DMM are possible. Within the framework of this paper, one may also consider a within-group model, in which the IRFs are assumed to be increasing only in 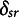 . Such a model may be useful if the focus is on within-group comparison only rather than comparison of all respondents, or if items contain a relative component in relation to a group aspect. Properties and applications of this model are yet unknown. Outside the framework proposed in this paper, Koopman, Zijlstra, De Rooij, and Van der Ark (2020) proposed the nonparametric hierarchical rater model, a nonparametric version of the (parametric) hierarchical rater model (Patz et al., 2002). Possibly other two-level parametric IRT models may be redefined as a nonparametric model, such as the multiple raters model (Verhelst & Verstralen, 2001) or the rater bundle model (Wilson & Hoskens, 2001). Alternatively, the nonparametric partial credit model or nonparametric sequential model (Hemker et al., 1997, 2001, respectively) may be generalized to a two-level framework.

The presented models in this paper are unidimensional models. Hence, for MHM-1 and DMM-1, it is assumed that respondents across groups may be located on the same latent variable. This is quite a strict assumption and whether this is sensible should be investigated, for example, by analysis on differential item functioning (Holland & Wainer, 1993). Known methods within nonparametric IRT are comparing scales and scale properties across groups (Sijtsma & Van der Ark, 2017; Van der Ark et al., 2008) and performing an IIO analysis (Sijtsma & Junker, 1996). Two-level IRT modeling may benefit from multidimensional generalizations for developing scales that explicitly separate a respondent and group dimension. How these alternative models hierarchically relate to the models presented in this paper, and what properties they imply, is a topic for further investigation.

The developments presented in this paper are part of a larger project to make all elements of Mokken scale analysis available for two-level test data (Koopman, Zijlstra, & Van der Ark, 2020; Koopman et al., 2022). Next steps in development should be aimed at developing group-level item selection procedures and at allowing more complex research designs, such as a cross nested design in which respondents score multiple groups.

## APPENDIX

Lemma A.1. UN-1 implies UN-2.
Proof. Equation 4 defines  , which implies  . However, as  does not depend on *r*,  , where  denotes the expectation of  over the respondents in randomly selected *s*. Because  , it follows that  within group *s*. If  is unidimensional, its expectation  is also unidimensional. Variable  is unidimensional by UN-1; hence, variable  is also unidimensional.
Lemma A.2. MO-1 implies MO-2.
Proof. Let  denote the probability density function of the distribution of  . By H, the group-level item-step response function is

(A.1)

As  and  are independent by B,  , and the last term of Equation A.1 reduces to

(A.2)

By MO-1,  is nondecreasing in  . Hence, Equation A.1 is nondecreasing in  , which equals the definition of MO-2.
Lemma A.3. IIO-1 implies IIO-2.
Proof. By IIO-1

(A.3)

The final result in Equation A.3 equals the definition of IIO-2.
Lemma A.4. MHM-1 implies that  is nondecreasing in  for any bounded, nondecreasing function  .
Proof. By LI-1, scores  within  are independent given  . By MO-1,  is stochastically ordered in  ; that is, for  ,  for all *i* and all *x*. For a set of independent variables the stochastic ordering is preserved under convolutions, for any bounded, nondecreasing function  (Shanthikumar e.g., 2007, Theorem 1.A.3(b); see also Ahmed et al. 1981, Lemma 3.3; Holland and Rosenbaum 1986, Lemma 2). Hence

(A.4)

Lemma A.5. MHM-2 implies  is nondecreasing in  for any bounded, nondecreasing function  .
Proof. Assumptions UN-2, LI-2, and MO-2 are equivalent to Rosenbaum’s (1988) assumptions (1), (6), and (7), respectively, which collectively define the item-bundel model. In his Lemma 1, Rosenbaum showed that for any bounded, nondecreasing function  for which UN-2, LI-2, and MO-2 holds,  is nondecreasing in  (see, also, Kamae et al., 1977, Proposition 1).
Proof of Theorem 1.
*(B implies LI-1 and LI-2)*
Proof. The independence of the  ,  , and  by B implies that the  are independent given  , and that the (  ,  ) are independent given  . This, combined with  , implies LI-1 and LI-2 in the following way. For each  , given  , the  are a function of  . Because for each  , the  are independent given  , the  are independent given  , and LI-1 is implied. Furthermore, the  are independent given  . Because  are a function of  , given  the  are a function of  . Hence,  are independent given  , and LI-2 is implied.
Proof of Theorem 2.
*(Under B and H, UN-1, MO-1, and IIO-1 imply UN-2, MO-2, and IIO-2, respectively.)*
Proof. First, we consider the extreme case of no respondent variance: If var  = 0, then  and  for all *r* and all *s*. As a result,  ,  , and  . Hence, UN-1 = UN-2 (Definiton 1), MO-1 = MO-2 (Definition 3), and IIO-1 = IIO-2 (Definition 4). Second, for var  , Lemma A.1 proves that UN-1 implies UN-2, Lemma A.2 proves that MO-1 implies MO-2, and Lemma A.3 proves that IIO-1 implies IIO-2.
Proof of Theorem 3.
*(For dichotomous item scores (a) the MHM-1 implies MLR-1 and (b) for*  *, the MHM-2 implies MLR-2.)*
Proof.

- The assumptions in MHM-1 are identical to the assumptions used by Grayson (1988, Theorem 2) and Huynh, 1994 to establish MLR of the sum score in  , hence their proof can be applied.
- For clarity, we give the proof for  , but it can straightforwardly be generalized for  . Let  be the number of ways that respondents  can be ordered, and let  be an index of possible respondent orderings. Furthermore, let  denote the the position of respondent *r* in respondent-ordering *d*. For  , the same method can be applied, but each permutation contains only *I* respondents, hence the number of permutations  .
  Let  denote the group-level sum score in which each item score is taken from a different respondent, with realization  . Let  denote the vector of item scores from the respondent order from the *d*th permutation, with realization  . For a given permutation *, let  denote the sum over all possible patterns of *I* item scores that sum to  . Let  and let  . By LI-2, for  , item scores  and  are independent conditional on  . Hence, for dichotomous items, the probability of obtaining group-level sum score  is
  (A.5)
  Because  is constant across each item-score pattern  , Equation A.5 is identical to
  (A.6)
  The form of the right-hand side in Equation A.6 is equal to the form used by Grayson (1988, Theorem 2) and Huynh, (1994). Hence, their methods can be applied to establish MLR of the sum score  in  . Because  ,
  (A.7)
  it follows that MLR also holds for  in  .

Proof of Theorem 4.
*((a) The MHM-1 implies SOM-1 and (b) the MHM-2 implies SOM-2.)*
Proof.

- SOM-1 follows from the general result presented in Lemma A.4. First, note that the respondent-level sum score  is a nondecreasing function of  (e.g., Rosenbaum, 1984). Hence, by Lemma A.4,  is nondecreasing in  , which is the definition of SOM-1.
- SOM-2 follows from the general result presented in Lemma A.5. First, note that the group-level sum score  is a nondecreasing function of  . Hence, by Lemma A.5,  is nondecreasing in  , which is the definition of SOM-2.

Proof of Theorem 5.
*((a) The MHM-1 implies WSOL-1 and (b) the MHM-2 implies WSOL-2.)*
Proof.

- MHM-1 implies SOM-1 of  by  (Theorem 4(a)). Van der Ark & Bergsma, (2010, Theorem) showed that SOM implies WSOL, hence WSOL of  by  is implied.
- Similar to the proof in (a), the MHM-2 implies SOM-2 of  by  , hence, WSOL of  by  is implied.

Proof of Theorem 6.
*(For dichotomous items (a) the MHM-1 implies MM-1 and (b) the MHM-2 implies MM-2.)*
Proof.

- The proof is analogous to the proof in Proposition 4.1a Junker, (1993). By the law of total expectation (e.g., Rice, 2006, p 149) and LI-1
  (A.8)
  For dichotomous items, under the MHM-1, by Theorem 3(a),  is nondecreasing in  (SOL). Because  is stochastically ordered in  , so is any nondecreasing function of  , such as  (Equation 6; Shaked & Shanthikumar 2007, Theorem 1.A.3.(a)), which completes the proof.
- This proof is parallel to the proof in (a), which holds when substituting  by  ,  by  ,  by  , LI-1 by LI-2, and MHM-1 by MHM-2.

Proof of Theorem 7.
*((a) The MHM-1 implies CA-1 and (b) the MHM-2 implies CA-2.)*
Proof.

- The proof is similar to the proof of Theorem 1 by (Rosenbaum, 1984; see also Holland and Rosenbaum (1986, Theorem 6). If CA-1 holds, the conditional covariance  (Definition 9). Using standard algebra, it can be shown that this statement is equivalent to
  (A.9)
  (e.g., Rice, 2006, p. 138). Hence, we prove that under the MHM-1 Equation A.9 holds. By the law of total expectation,
  (A.10)
  (e.g., Rice, 2006, p. 138). By LI-1,  and  are independent given  . Hence,
  (A.11)
  Because, by LI-1, the values in  are independent given  , they are associated (Esary et al., 1967, Theorem 2.1). Therefore,
  (A.12)
  By Lemma A.4,  and  are nondecreasing in  , hence, they are associated (Esary et al., 1967,  ). In addition, by UN-1,  is a scalar and therefore associated (Esary et al., 1967,  ). Hence, it follows that
  (A.13)
  By the law of total expectation, the statement in Equation A.13 is equivalent to
  (A.14)
  which completes the proof.
- The proof is similar to the proof in (a). Throughout the proof,  is kept the same, but  is replaced by  , with  . Hence,  and  apply to different respondents within the same group. Furthermore,  is replaced by  , hence to the vector that contains all item scores in group s, except the scores of respondents *r* and *p*. Finally,  is replaced by  , LI-1 by LI-2, and Lemma A.4 by Lemma A.5, which gives the proof for (b) (see, also, Rosenbaum, 1988).

Proof of Theorem 8.
*((a) The DMM-1 implies MIIO-1 and (b) the DMM-2 implies MIIO-2.)*
Proof.

- This proof is similar to the proof of the Corollary by Ligtvoet et al., (2011). By IIO-1,  . By Equation 5,  equals
  (A.15)
  By LI-1,  , and  are independent given  , and their joint probability equals the product of their marginal conditional probabilities (e.g., Rice, 2006, p. 84). Hence, Equation A15 equals
  (A.16)
  Let  denote the cumulative distribution function of  . Integrating both sides of Equation A.16 over  yields
  (A.17)
  for all *y* and all  , completing the proof.
- The proof is parallel to the proof in (a). Replacing  by  ,  by  , IIO-1 by IIO-2, LI-1 by LI-2,  by  , proofs that the MHM-2 implies MIIO-2.
